# Recombinant self-assembling peptides as biomaterials for tissue engineering

**DOI:** 10.1016/j.biomaterials.2010.08.051

**Published:** 2010-12

**Authors:** Stuart Kyle, Amalia Aggeli, Eileen Ingham, Michael J. McPherson

**Affiliations:** a Institute of Molecular and Cellular Biology, Faculty of Biological Sciences, University of Leeds, LS2 9JT, UK; b Astbury Centre for Structural Molecular Biology, Faculty of Biological Sciences, University of Leeds, LS2 9JT, UK; c Centre for Molecular Nanoscience, School of Chemistry, University of Leeds, LS2 9JT, UK; d Institute of Medical and Biological Engineering, University of Leeds, LS2 9JT, UK

**Keywords:** Recombinant expression, Self-assembly, Peptide, Hydrogel, Cytocompatibility

## Abstract

Synthetic nanostructures based on self-assembling systems that aim to mimic natural extracellular matrix are now being used as substrates in tissue engineering applications. Peptides are excellent starting materials for the self-assembly process as they can be readily synthesised both chemically and biologically. P_11_-4 is an 11 amino acid peptide that undergoes triggered self-assembly to form a self-supporting hydrogel. It exists as unimers of random coil conformations in water above pH 7.5 but at low pH adopts an antiparallel β-sheet conformation. It also self-assembles under physiological conditions in a concentration-dependent manner. Here we describe an unimer P_11_-4 production system and the use of a simple site-directed mutagenesis approach to generate a series of other P_11_-family peptide expression vectors. We have developed an efficient purification strategy for these peptide biomaterials using a simple procedure involving chemical cleavage with cyanogen bromide then repeated filtration, lyophilisation and wash steps. We report peptide-fusion protein yields of ca. 4.64 g/L and we believe the highest reported recovery of a recombinant self-assembling peptide at 203 mg/L of pure recombinant P_11_-4. This peptide forms a self-supporting hydrogel under physiological conditions with essentially identical physico-chemical properties to the chemically synthesised peptide. Critically it also displays excellent cytocompatibility when tested with primary human dermal fibroblasts. This study demonstrates that high levels of a series of recombinant self-assembling peptides can be purified using a simple process for applications as scaffolds in tissue engineering.

## 1 Introduction

Over the past two decades self-assembling peptides have emerged as potential candidates for the development of safe nanostructured scaffolds in the field of tissue engineering [1–10]. Oligomeric peptides have been rationally designed to spontaneously self-assemble into hierarchical structures in solution, in response to specific physico-chemical environmental triggers, thus providing a new generation of well-defined biopolymers [11–14]. Under controlled conditions self-assembling peptides of the P_11_-family undergo one-dimensional self-assembly, forming single molecule thick, micrometer-long β-sheet nanotapes. Further assembly results in the nanotapes stacking in pairs to form ribbons which further assemble to form fibrils, then pairs of fibrils can entwine to form fibres. Over twenty systematically varied P_11_ peptides have been designed by Aggeli and colleagues to self-assemble using various physico-chemical triggers and give rise to self-supporting isotropic or liquid crystalline hydrogels at peptide concentrations of 10–30 mg/ml [12]. These peptides vary in terms of overall charge, hydrophobicity and type of polar amino acids resulting in nanostructured fibrillar peptide networks with different solvent affinity and dissolution rate.

**Fig. 1:**
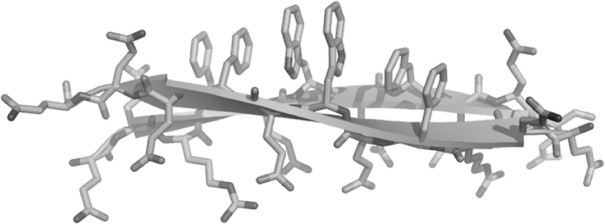
A schematic representation of rP_11_-4 peptide (QQRFEWEFEQQ) adopting an antiparallel β-sheet conformation.

As an example, self-assembling peptide P_11_-4 (QQRFEWEFEQQ) (Fig. 1) was designed to form fibrils at low pH and to be monomeric in solution at higher pH. The incorporation of glutamic acid residues at position 5, 7 and 9 means there is no charge on the γ-carboxyl groups at pH ≤2. However a positive net charge of one exists on the arginine. This allows antiparallel β-sheet assembly by Coulombic interactions. The terminal glutamine residues contribute to hydrophobic interactions as well as complementary hydrogen bonding, whilst the aromatic residues phenylalanine, tryptophan and phenylalanine at positions 4, 6 and 8 respectively, promote π–π interactions of the aromatic rings and aid in the association of tapes into ribbons in aqueous conditions. By contrast at higher pH, all three glutamates carry a negative charge and electrostatic repulsion will therefore prevent efficient β-sheet formation. Furthermore, the addition of NaCl to a concentration of 140 mm has been shown to induce a fluid to gel transition at a critical concentration of 6.3 mm at pH 6. The sodium ions will screen the electrostatic forces between the net negatively charged P_11_-4 fibrils [12].

Most self-assembling peptides described in the literature have been produced using solid-state synthesis. This is efficient for small scale production and testing, but modest scale-up can be expensive while industrial scale production can be very challenging. It can also be difficult to produce peptides of lengths over 35 residues in acceptable yields and there are environmental considerations in disposal of chemical waste. An alternative approach is to produce peptides biosynthetically using recombinant microorganisms [15], which presents different challenges. Specifically, such small peptides are often difficult to express in bacterial cells due to their propensity to self-associate, mediate toxicity or be rapidly degraded by proteases [16]. Such issues can be addressed by fusing the peptide to a larger carrier protein to improve intracellular stability [17] and provide a mechanism for initial purification. A further problem then arises in removing the fusion partner, following chemical or enzymatic cleavage, to leave pure peptide monomer in solution.

We have previously reported on the use of a pET31-based expression system to produce trimers of P_11_-4 with subsequent cyanogen bromide cleavage and reverse phase HPLC [18]. This study provided the highest yields of recombinant self-assembling peptide-fusion protein (2.5 g/L) using an auto-induction process [19] and of pure peptide (90 mg/L). However, one issue with this trimer approach was the production of peptide species with a C-terminal homoserine lactone moiety due to cyanogen bromide (CNBr) cleavage. Similar studies have been reported by Middelberg and co-workers on recombinant production of a related peptide P_11_-2 (QQRFQWQFEQQ) using a pET31 system with intervening cysteines and cleavage of peptides by 1-cyano-4-dimethylaminopyridinium tetrafluoroborate. In this case protein expression used IPTG induction and therefore gave relatively low levels of protein with purification of 2.63 mg peptide/L culture after reverse phase (rp)HPLC which represents a 42% yield of peptide [17]. Subsequently they developed a chromatography-free purification system based on thioredoxin as the fusion partner and with tobacco etch virus protease release of the peptide in heat treated whole cell homogenates. Ethanol precipitation in the presence of 250 mm NaCl was used to precipitate other material leaving peptide in solution. They recovered 13 mg peptide/L culture, although they did use a rpHPLC step to perform buffer exchange of peptide samples for characterisation [20].

The purpose of this study was to improve upon a recombinant expression and purification strategy [18] for self-assembling peptide production, to simplify the purification, to demonstrate the general applicability of the approach for peptide biomaterial production and to perform more detailed characterisation (hydrogel formation, CD, FTIR, TEM) and cytocompatibility studies on one of these recombinant peptides, P_11_-4 (rP_11_-4). To ensure production of homogenous native peptide without any associated homoserine lactone species derived from CNBr cleavage, we used an unimer fusion construct with the ketosteroid isomerase separated from a single P_11_-4 peptide encoding sequence by a methionine residue. We then used this recombinant vector as a template to generate a series of further P_11_-family peptide constructs by using a site-directed mutagenesis strategy. To simplify the downstream processing we used cell lysis and fractionation to give the insoluble inclusion body-based pellet, with subsequent cleavage of the peptide from the fusion protein, and non-chromatographic purification of peptide in water. All the purified peptides were analysed by SDS-PAGE and mass spectrometry. Purified rP_11_-4 was then used directly in further peptide characterisation assays and importantly the cytocompatibility of rP_11_-4 hydrogel was also tested using primary human dermal fibroblasts.

## 2 Materials and methods

### 2.1 Bacterial strains, plasmids, and cell culture medium

*Escherichia coli* XL1 Blue (Stratagene, La Jolla, CA.) was used for routine cloning and BL21 Star (DE3) (Invitrogen, Carlsbad, CA.) was used as the expression strain. The expression vector pET31b+ was from Novagen, UK.

The growth medium Luria Burtani (LB), and LB agar were prepared according to [21]. Auto-induction medium was prepared as described by [19]. The components of various auto-induction media are given in Table 1. Carbenicillin (Fisher Scientific Ltd., UK) was added to medium to a final concentration of 100 μg/ml.

### 2.2 Generation of recombinant *E. coli* strains

**Fig. 2:**
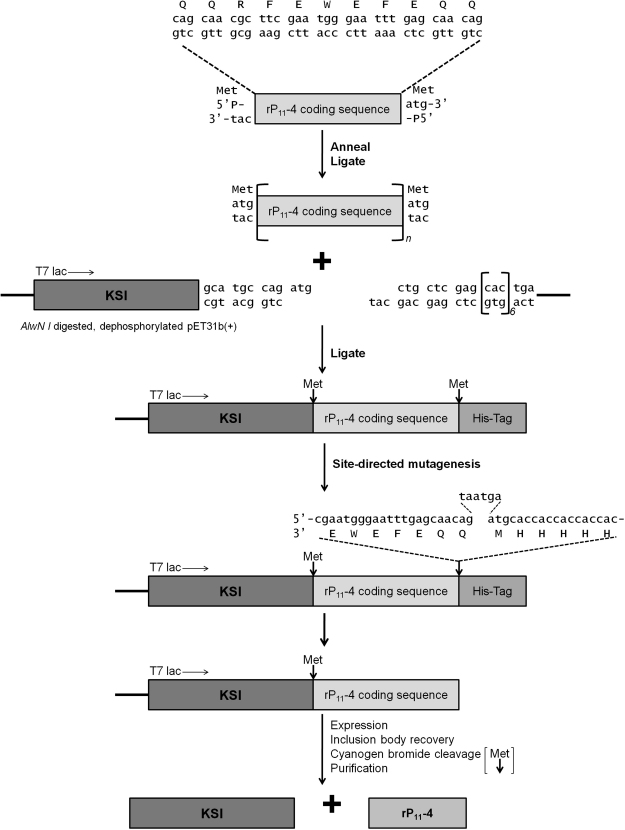
Cloning and expression strategy for rP_11_-4 peptide production. P_11_-4 encoding oligonucleotide sequences were designed to introduce intervening methionine codons (ATG) when annealed and cloned into the *Alw*NI site of pET31b. Site-directed mutagenesis was used to introduce two termination codons (TAATAG) to prevent translation of the C-terminal methionine and hexa-histidine tag. KSI-rP_11_-4 fusion protein was expressed and individual rP_11_-4 monomers cleaved from the insoluble KSI using CNBr generating a mixture of KSI and rP_11_-4. rP_11_-4 unimers were purified by adjusting the pH to 9, then filtration, lyophilisation and wash cycles.

The design of rP_11_-4 encoding DNA and cloning into pET31b+ has previously been described [18]. Site-directed mutagenesis using a Quikchange protocol was used to introduce two stop codons (bold) before the sequence encoding the C-terminal hexa-histidine tag in pET31b-KSI-rP_11_-4(1)-HIS (Fig. 2; [18]) using the following primers.

F: 5′-CGAATGGGAATTTGAGCAACAG**TAATAG**CTCGAGCACCACCACCACCAC-3′;

R: 5′-GTGGTGGTGGTGGTGCTCGAG**CTATTA**CTGTTGCTCAAATTCCCATTCG-3′.

A 50 μl reaction volume comprising 1 μl pET31b-KSI-rP_11_-4(1)-HIS (10 ng), 1.5 μl (10 pmol/μl) of each primer, 5 μl 2 mm dNTPs, 5 μl 10 × KOD Hot Start DNA polymerase buffer (200 mm Tris–HCl, 80 mm MgCl_2_, 5 mm DTT, 500 mg/ml BSA, pH 7.5), 3 μl 10 mm MgSO_4_, 1 μl KOD Hot Start DNA polymerase (1 U) and 32 μl water (molecular biology grade) was subjected to: 1 min at 94 °C followed by 18 cycles of 30 s at 94 °C, 30 s at 55 °C and 8 min at 68 °C and a final extension step at 72 °C for 20 min. The reaction products were treated with *Dpn* I (1 U) for 1 h to digest the methylated template DNA.

*E. coli* XL1 Blue cells were thawed on ice prior to addition of 20 ng *Dpn* I digest. After 15 min on ice the sample was incubated at 42 °C for 90 s and then on ice for 2 min. LB medium (1 ml) was added and the sample incubated at 37 °C in an orbital incubator (250 rpm) for 1 h. Transformed cells (50 μl) were then plated onto LB agar with carbenicillin (100 μg/ml) and incubated at 37 °C for 16 h.

Colonies were cultured in 5 ml LB medium with carbenicillin (100 μg/ml) in an orbital incubator (250 rpm) at 37 °C for 16 h. The cells were harvested by centrifugation at 4000 *g* for 10 min at 4 °C and used for plasmid DNA purification using a QIAprep MiniPrep Kit (Qiagen Ltd., UK) according to the manufacturer’s instructions. The pET31b-KSI-rP_11_-4(1) DNA was eluted in 50 μl 10 mm Tris.HCl, pH 8.5 and stored at −20 °C and the DNA sequence of the insert region confirmed using T7 forward and reverse primers (ABI 3130xl capillary sequencer, DNA Sequencing Facility, Faculty of Biological Sciences, University of Leeds).

To create further self-assembling peptide variants P_11_-2, P_11_-9, P_11_-13, P_11_-14, P_11_-20 and P_11_-4+RGD, site-directed mutagenesis as described above was performed on the pET31b-KSI-rP_11_-4(1) plasmid using the following primers:

KSI-rP_11_-2-F GCCAGATGCAGCAACGCTTTCAGTGGCAGTTTGAGCAACAGTAATAGC
KSI-rP_11_-2-R GCTATTACTGTTGCTCAAACTGCCACTGAAAGCGTTGCTGCATCTGGC
KSI-rP_11_-9-F AGCAGCCGCTTTGAATGGGAATTTGAAAGCAGCTAATAGCTCGAGCAC CACCAC
KSI-rP_11_-9-R GCTGCTTTCAAATTCCCATTCAAAGCGGCTGCTCATCTGGCATGCGT GAATATTC
KSI-rP_11_-13-F GAACAGGAATTTGAATGGGAATTTGAACAAGAGTAATAGCTCGAG CACCACCAC
KSI-rP_11_-13-R CTCTTGTTCAAATTCCCATTCAAATTCCTGTTCCATCTGGCATGCG TGAATATTC
KSI-rP_11_-14-F AAATTTAAATGGAAATTTAAGCAACAGTAATAGCTCGAGC
KSI-rP_11_-14-R TTAAATTTCCATTTAAATTTTTGCTGCATCTGGCATGCG
KSI-rP_11_-20-F GCCAGATGCAGCAACGGCAGGAACAGGAACAGGAGCAACAGT AATAGCTCG
KSI-rP_11_-20-R CGAGCTATTACTGTTGCTCCTGTTCCTGTTCCTGTTCCTGGCGT TGCTGCATCTGGC
KSI-rP_11_-4+RGD-F CGCGGTGATGGCGGTGGCGGTCAGCAACGCTTCGAATGGG
KSI-rP_11_-4+RGD-R ACCGCCACCGCCATCACCGCGCSTCTGGCATGCGTGAATATTC

Plasmid DNA was purified and DNA sequencing was used to confirm the correct sequences of each construct. These plasmids are similarly designated pET31b-KSI-rP_11_-N(1) where N corresponds to the peptide number, so for example the construct encoding P_11_-20 is designated pET31b-KSI-rP_11_-20(1).

### 2.3 Protein expression

The protocols developed by Studier [19] were followed. *E. coli* BL21 Star (DE3) strains carrying expression plasmids were inoculated into 2 ml LB medium with carbenicillin (100 μg/ml) and grown in an orbital incubator (250 rpm) at 37 °C for 6 h. A 200 μl aliquot of the starter culture was used to inoculate 400 ml auto-induction medium in a 2 L baffled flask with growth at 25 °C in an orbital incubator (250 rpm) for up to 72 h. Aliquots (1 ml) of cells were removed at time points between 16 and 72 h and harvested at 13 000 rpm in a microcentrifuge. At 72 h the remaining culture volume was harvested by centrifugation at 5000 rpm in a fixed angle rotor (Sorvall SLA3000) for 15 min.

### 2.4 Preparation of soluble and insoluble proteins from *E. coli* cultures

Cells were lysed with lysis buffer (50 mm HEPES, 25% (w/v) sucrose, 5 mm MgCl_2_.6H_2_O and 1% (v/v) Triton X-100; pH 8). The pH of the solution was adjusted to pH 8 using 6 m HCl or 6 m NaOH. Immediately prior to use, Omnicleave™ endonuclease (2 μl per 40 ml lysis buffer; Epicentre, Cambridge, UK) was added to the lysis buffer, together with lysozyme to 1 mg/ml. The lysate was sonicated at 20 kHz using 4 bursts of 20 s with 20 s intervals then centrifuged at 22,000 *g* for 30 min to separate the soluble and insoluble material. Aliquots of the supernatant (soluble protein fraction) were reserved for analysis. The pellet containing the inclusion bodies was washed 4–5 times in 0.1× lysis buffer by resuspension and centrifugation at 10,000 *g*. The resulting pellets were stored at −20 °C until required.

### 2.5 SDS-PAGE analysis

SDS-PAGE was used to separate soluble and insoluble protein fractions. An XCell *SureLock*™ mini-cell system (Invitrogen, Ltd.) was used with NuPAGE 4–12% Bis-Tris precast gels (Invitrogen, Ltd.). Samples were prepared in loading buffer (Invitrogen, Ltd.) and heated at 95 °C for 5 min prior to gel loading. Gels were electrophoresed at a constant voltage of 190 V for 40 min then stained with SimplyBlue™ Safestain (Invitrogen, Ltd.) with gentle agitation for 1 h at room temperature and washed several times in double distilled water for 24 h. Gel images were acquired using a Kodak Gel Logic 1500 system (Kodak Ltd.).

### 2.6 Protein concentration determination

Purified protein concentrations were determined by the Bradford assay (Sigma–Aldrich, Ltd.). Peptide concentrations were determined by A_280_ measurement and concentrations were calculated using the Beer–Lambert Law, taking the molar extinction coefficient (*ε*) of rP_11_-4 as 5500 m^−1^ cm^−1^ [22].

### 2.7 Cleavage of the KSI fusion protein by cyanogen bromide (CNBr)

The KSI-r(peptide) fusion proteins were resuspended at a concentration of 10 mg/ml in 70% (w/v) formic acid. CNBr was dissolved in 70% (w/v) formic acid and added to the protein sample to provide a 200-fold molar excess of CNBr to methionine residues. The flask was purged with N_2_ and incubated in the dark for 24 h with gentle agitation. The formic acid was then removed by rotary evaporation until a gelatinous material formed.

### 2.8 Purification of CNBr cleaved peptide

This gelatinous material was resuspended in 40 ml double distilled water and the pH adjusted to 9 using either 5 m NaOH or 7 N ammonia in methanol. The soluble and insoluble fractions were separated by centrifugation at 10,000 *g* for 30 min. The supernatant was filtered through a 0.22 μm nitrocellulose filter that was prewashed with water at pH 9. The filtrate was lyophilised and the resulting powder resuspended in 10 ml double distilled water and agitated for 2 h to allow the peptide to fully dissolve. The process of wash, filtration and lyophilisation was repeated five times. A final lyophilisation was performed by snap freezing the samples in liquid nitrogen which were then placed into a Heto LyoPro 6000 freeze drier until a dry powder was formed.

### 2.9 Mass spectrometry

Protein and peptide samples were prepared for electrospray ionisation mass spectrometry (ESI-MS) by dissolving dry powders in 20 μl formic acid. Samples were submitted to the Mass Spectrometry Facility, Astbury Centre, University of Leeds and analysed by Dr. J. Ault on a Synapt HDMS (Waters UK Ltd.) mass spectrometer. Peptide samples were subsequently sequenced by tandem mass spectrometry (MSMS).

### 2.10 Formation of rP_11_-4 peptide hydrogel

Lyophilised rP_11_-4 (10 mg/ml) was reconstituted in either cell culture medium (DMEM) or 140 mm NaCl in water (pH 7.4). In order to monomerise and reverse self-assembly of rP_11_-4 the pH was increased to 9 using 0.5 m NaOH.

### 2.11 Transmission electron microscopy

rP_11_-4 was prepared at a concentration of 10 mg/ml (6.3 mm) in 140 mm NaCl (pH 7.4) and diluted to a peptide concentration of 100 μm in water. Glow discharged, carbon-coated 400 hexagonal mesh copper grids were covered with 20 μl peptide solution and allowed to adsorb for 1 min. Excess sample was removed using filter paper and grids were negatively stained by the addition of 10 μl 2% (v/v) uranyl acetate in water for 20 s. Excess stain was removed with filter paper and grids were air dried prior to analysis. Images were obtained using a Jeol 1200 EX TEM operating at 80 kV.

### 2.12 Circular dichroism UV spectroscopy of rP_11_-4

Lyophilised rP_11_-4 hydrogel was prepared at a concentration of 10 mg/ml (6.3 mm) in 140 mm NaCl. The pH was adjusted to 7.4 in which peptide fibril association occurred and pH 9 in which peptide fibril dissociation occurred. Samples were left to equilibrate at 25 °C for 24 h and diluted in distilled water to a final concentration of 100 μm prior to analysis. The pH was then reconfirmed. Samples were then placed in quartz cuvettes (Hellma^®^) with a path length of 0.1 mm. Mean residual ellipticity readings were taken in the far UV region of the spectrum, (190–240 nm) using a Jasco J-750 spectropolarimeter. Each spectrum was the average of five scans with a step resolution of 0.5 nm, scan speed 50 nm min^−1^, response time of 1 s and a sensitivity of 50 m° at 20 °C. Blank readings were taken for all samples and subtracted from the data obtained from the peptide samples.

### 2.13 Fourier-transform infra-red spectroscopy analysis of rP_11_-4

rP_11_-4 was prepared by dissolving 10 mg of lyophilised peptide in 1 ml of 140 mm NaCl in D_2_O. The pH of the solution was adjusted by addition of hydrochloric acid (DCl) or sodium hydroxide (NaOD) to pH 7.4 and 9 respectively. Samples were mixed thoroughly for 1 min and left to equilibrate at 25 °C for 24 h. Samples (50 μl) were then placed between CaF_2_ cells separated by a 50 μm Teflon spacer. Spectra obtained were averages of four scans, recorded with a resolution of 4 cm^−1^ at 25 °C using a Nicolet Magna 560 FTIR spectrometer, in which blank solvent spectra were subtracted.

### 2.14 Contact cytotoxicity assay

Samples of rP_11_-4 hydrogel (*n* = 3) were attached to the centre of each well of six-well tissue culture plates using collagen type I extracted from rat tail tendons (acid solubilised rat tail collagen and neutralised using 0.1 m NaOH). As a positive control cyanoacrylate glue was placed into the centre of each well of a 6-well tissue culture plate, as it has been demonstrated to produce a reproducible cytotoxic response. As the negative control collagen type I was placed into the centre of each well of a 6-well plate as it has been demonstrated to be biocompatible and thus not cytotoxic. Primary human dermal fibroblasts (passage 3, Cascade Biologics, Nottingham) were seeded at a density of 5 × 10^4^ cells/ml and DMEM plus supplements (10% (v/v) fetal calf serum, 100 U/ml penicillin, 100 mg/ml streptomycin, 10 μm l-glutamine) added to a total of 2 ml. Plates were incubated at 37 °C in 5% (v/v) CO_2_ in air for 48 h. The culture medium was then carefully aspirated before the wells were washed in phosphate buffered saline (PBS). Each well was fixed using 10% (v/v) neutral buffered formalin for 10 min then stained with Giemsa solution (Merck R66 formulation) for 5 min. The wells were then repeatedly rinsed with distilled water and the plate allowed to air dry at 25 °C. The dry plates were examined by light microscopy to view any changes in cell morphology and confluence.

## 3 Results

### 3.1 Cloning and expression strategy

The cloning and expression strategy adopted for generating the unimer P_11_-4 construct is shown in Fig. 2. The plasmid pET31b-KSI-rP_11_-4(1)-HIS was previously generated [18]. This plasmid contained a single P_11_-4 coding sequence, but has the C-terminal Met and 6His tag which were not required in this study. Site-directed mutagenesis was used to insert two translation termination codons upstream of the C-terminal Met to prevent expression of this Met or the 6His tag so that CNBr cleaved product would result in release of a native sequence peptide from the KSI partner. The correct sequence was confirmed by DNA sequencing and designated pET31b-KSI-rP_11_-4(1).

We then used this vector as a template for site-directed mutagenesis to create a series of plasmids encoding other members of the self-assembling peptide P_11_-family fused to the KSI gene in the pET31b vector. The series of constructs generated encoded P_11_-2, P_11_-4, P_11_-9, P_11_-13, P_11_-14, P_11_-20 and P_11_-4+RGD.

### 3.2 Expression of KSI-rP_11_-N from *E. coli* BL21 Star (DE3) by auto-induction

**Fig. 3:**
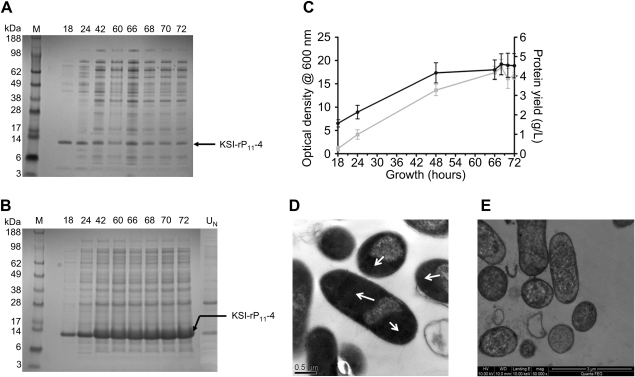
Expression of KSI-rP_11_-4 fusion protein. SDS-PAGE analysis of KSI-rP_11_-4 expressed in *E. coli* BL21 (DE3) Star cells by auto-induction from 18 to 72 h in (A) soluble and (B) insoluble fractions. U_N_, uninduced control in which no lactose was added to the culture medium and grown for 72 h. (C) Changes in culture densities () and protein yields () over time. Data expressed as (*n* = 3) ± 95% confidence limits. TEM analysis of *E. coli* cells carrying a pET31b-KSI-rP_11_-4 construct (D) induced showing dense inclusion body formation and (E) uninduced showing no inclusion body formation.

The pET31b-KSI-rP_11_-N(1) plasmids were transformed into *E. coli* BL21 Star (DE3) cells for expression trials. Initially cultures expressing the KSI-P_11_-4 fusion protein were grown in 8ZY4Lac auto-induction medium (Table 1) for up to 72 h with time course samples taken at regular intervals. Soluble and insoluble cell fractions were analysed by SDS-PAGE as shown in Fig. 3A and B. The KSI-(P_11_-4) fusion protein was predominantly found in the insoluble fractions. The highest culture densities at OD_600_ of 18.8 were reached after 70 h of growth (Fig. 3C). Insoluble protein yields were determined to be at an optimum of 4.64 g/L when bacterial cells were grown in 8ZY4Lac and harvested after 70 h growth. Auto-induction expression cultures for the other pET31b-KSI-P_11_-N(1) constructs in BL21 Star (DE3) were therefore subsequently grown for 70 h before harvesting.

### 3.3 Visualisation of cytoplasmic inclusion bodies by TEM in *E. coli* BL21 Star (DE3) cells expressing KSI-rP_11_-4

TEM was used to visualise electron dense inclusion bodies within the cytoplasm of *E. coli* BL21 (DE3) Star cells after 48 h auto-induction. TEM images showed electron dense areas at the poles of the cells, which is typical of protein based inclusion bodies found in the cytoplasm of *E. coli* cells (Fig. 3D). The negative control of *E. coli* cells containing no plasmid showed heavy staining from uranyl acetate yet no dense areas located at the poles of the cells were observed (Fig. 3E).

### 3.4 Preparation of soluble and insoluble proteins from *E. coli* cultures

Cells were lysed with lysis buffer 50 mm HEPES, 25% (w/v) sucrose, 5 mm MgCl_2_.6H_2_O and 1% (v/v) Triton X-100. The pH of the solution was adjusted to pH 8 using 6 m HCl or 6 m NaOH. Immediately prior to use, Omnicleave™ endonuclease (2 μl per 40 ml lysis buffer; Epicentre, Cambridge, UK) was added to the lysis buffer, together with lysozyme to 1 mg/ml. The lysate was sonicated on ice at 20 kHz using 4 bursts of 20 s on and 20 s off, then centrifuged at 22,000 *g* for 30 min to separate the soluble and insoluble material. Aliquots of the supernatant (soluble protein fraction) were reserved for analysis. The pellet containing the inclusion bodies was washed 4–5 times in 0.1× lysis buffer by resuspension and centrifugation at 10,000 *g*. The resulting pellets were stored at −20 °C until required.

### 3.5 Cyanogen bromide cleavage of KSI-P_11_-4 and rP_11_-4 purification

**Fig. 4:**
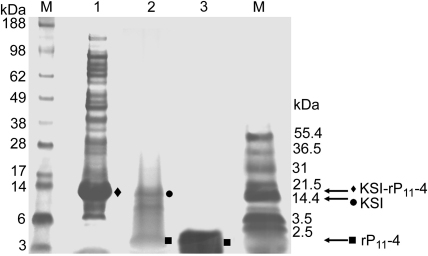
SDS-PAGE analysis of purification of rP_11_-4 showing intact fusion protein KSI-rP_11_-4 (♦, lane 1) before CNBr cleavage, CNBr cleavage products showing the KSI protein (●, lane 2), and purified rP_11_-4 using wash, filtration and lyophilisation steps (■, lane 3). The size (kDa) of molecular weight protein markers are indicated by M.

The process of cleavage and recovery of recombinant peptide was initially optimised for rP_11_-4. CNBr was used to cleave the inclusion body-based KSI fusion protein to release rP_11_-4. CNBr was added to a 200-fold molar excess of methionine residues to protein in 70% (v/v) formic acid in an oxygen free environment for 24 h, in the dark. The cleaved mixture was resuspended in double distilled water and the pH adjusted to 9 with ammonia. This should lead to recovery of rP_11_-4 in an unimeric state in solution. The mixture was centrifuged at 10,000 *g* for 30 min. We investigated the potential for purification of rP_11_-4 from the supernatant without the use of a chromatography step such as rpHPLC. The supernatant, at pH 9, was passed through a 0.22 μm filter, lyophilised and resuspended in 10 ml double distilled water (pH 9) and agitated for 2 h so that the peptide fully dissolved, then lyophilised. This process of resuspension at pH 9, filtration and lyophilisation was repeated five times and the resulting powder was resuspended in 2 ml double distilled water (pH 9) and a sample electrophoresed together with uncleaved and cleaved fusion protein, as shown in Fig. 4. It can be clearly seen that an apparently pure peptide product was obtained after these wash steps. It was essential to ensure that this simple protocol for producing pure peptide resulted in material that was compatible with human cell growth which is addressed later.

### 3.6 Cyanogen bromide cleavage of KSI-P_11_-N and rP_11_-N purification

**Fig. 5:**
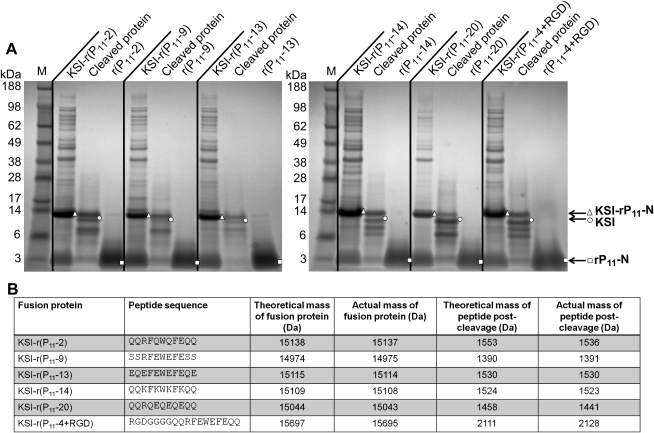
(A). SDS-PAGE analysis of KSI-P_11_-N fusion proteins before CNBr cleavage (KSI-rP_11_-N (N is the peptide number)), post-CNBr cleavage (cleaved protein), and purified peptide (rP_11_-N) recovered from the wash, filtration and lyophilisation steps in water at pH 9. Arrows indicate the various protein/peptide species (▵, KSI-rP_11_-N; ○, KSI; □, rP_11_-N). M, low molecular weight protein markers. (B) Sequence information, and theoretical and mass spectrometry determined mass data for the fusion proteins and purified peptides.

The cleavage and peptide purification procedure described above for rP_11_-4 was subsequently applied to the inclusion body pellets recovered from the cultures of the other KSI-P_11_-N samples. Fig. 5A shows the results of an SDS-PAGE analysis of the uncleaved, cleaved and purified peptide samples for the P_11_-2, P_11_-9, P_11_-13, P_11_-14, and P_11_-4+RGD series. As observed for P_11_-4 (Fig. 4) it is clear that CNBr cleavage has occurred and that apparently pure peptide samples (Fig. 5A) were obtained after the series of wash, filtration and lyophilisation steps. This result indicates that the production and simple non-chromatographic purification strategy is of general use for these self-assembling peptides.

### 3.7 Mass spectrometry characterisation

To confirm the identity of the fusion protein and purified rP_11_-4, mass spectrometry was performed. The theoretical molecular mass of KSI-P_11_-4 fusion protein determined using http://www.expasy.ch/tools/protparam.html was 1,5140 Da and that obtained by mass spectrometry was 1,5139.77 Da, which was in excellent agreement. Mass spectrometry identified three main species within the rP_11_-4 sample, the most abundant was 1537.7 Da with +17 Da species at 1553.6 Da and 1570.7 Da. Terminal glutamines that are not chemically blocked are prone to cyclization to form pyroglutamic acid with the loss of ammonia [23]. Given the theoretical molecular mass of rP_11_-4 at 1554.6 Da, the species at 1553.6 would correspond to rP_11_-4, perhaps as a deprotonated species. Loss of ammonia from the N-terminal glutamine would give a 1537.6 Da species consistent with the observed 1537.7 Da species. It seems most likely that the 1570.7 species was due to an ammonia adduct due to the use of ammonia to adjust the pH. MSMS analysis confirmed the identity of the peptide by identifying 8 of the 11 amino acids as N-…RFEWEFEQ…-C of rP_11_-4.

The other purified P_11_-N peptides were also characterised by mass spectrometry and Fig. 5B provides the values for expected and determined molecular masses for both the KSI fusion proteins and for the purified peptides. The determined masses are in good agreement with the expected values.

Peptide rP_11_-4 was then subjected to a series of further characterisation and cytocompatibility studies as described in the following sections.

### 3.8 rP_11_-4 peptide quantification

UV spectrophotometry was used to quantify rP_11_-4 as we have reported previously [18] by using the method proposed by [22] in which the concentration (M) was determined from A_280_ (1 mg ml^−1^; 1 cm) = 5500/M, where 5500 is the molar extinction coefficient of rP_11_-4.

The KSI-P_11_-4 fusion protein has 137 amino acids and rP_11_-4 consisted of 11 amino acids, therefore rP_11_-4 comprises 8% of the fusion protein. The maximum yield of fusion protein obtained by auto-induction (8ZY4Lac) was 4.64 g/L, which theoretically equates to 371.2 mg/L of rP_11_-4. Following cleavage with CNBr, the pH change, and several lyophilisation and wash steps in double distilled water, the concentration of rP_11_-4 recovered equated to 203 mg/L which corresponds to a 55% yield. While further optimisation is required in relation to purification efficiency, we have achieved the highest levels of fusion protein and peptide recovery so far reported in the literature without any chromatographic step (Table 2).

### 3.9 Formation of a self-supporting rP_11_-4 peptide hydrogel in physiological medium

**Fig. 6:**
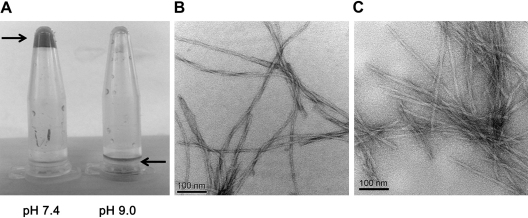
(A) Formation of rP_11_-4 10 mg/ml self-supporting hydrogel in DMEM cell culture medium. A hydrogel formed under physiological conditions (pH 7.4, 140 mm NaCl) while a pH shift to 9 resulted in peptide unimerisation and a gel–liquid transition. The black arrows indicate the position of the peptide sample. TEM analysis of uranyl acetate negative stained samples of (B) rP_11_-4 and (C) cP_11_-4 revealed similar semi-rigid fibril morphologies with the presence of fibrillar twists and bundles.

As has been previously demonstrated [24] cP_11_-4 formed a self-supporting hydrogel at a concentration of 10 mg/ml (6.4 mm) in cell culture medium (DMEM) at pH 7.4. In this study, rP_11_-4 purified by the lyophization and wash steps was resuspended in DMEM with the addition of 140 mm NaCl to a final concentration of 10 mg/ml. The pH of the solution was adjusted to 9 using NaOH, and to pH 7.4 using HCl. At pH 7.4 a self-supporting hydrogel formed, whereas at pH 9 no hydrogel formed and the sample remained in solution as shown in Fig. 6A.

### 3.10 Characterisation of rP_11_-4 fibril formation

Aggeli and colleagues have shown that cP_11_-4 forms a self-supporting hydrogel in 140 mm NaCl [24]. rP_11_-4 was diluted from 10 mg/ml in 140 mm NaCl and adjusted to 100 μm (0.156 mg/ml) at pH 2 in double distilled water. The resulting fibril morphologies can be seen by TEM using uranyl acetate negative staining in Fig. 6B. These images show that rP_11_-4 formed fibrils similar to cP_11_-4 (Fig. 6C). The fibrils were noted to be interconnecting with some twists being evident. It was also evident that some of these fibrils were long and curly, as well as forming a mesh-like appearance, whereas others were long and straight. Neighbouring fibrils can be seen as well as fibriller bundles and entanglements. Rope-like structures were also evident where individual fibrils started to elongate and wrap around each other.

### 3.11 Characterisation of rP_11_-4 peptide secondary structure

**Fig. 7:**
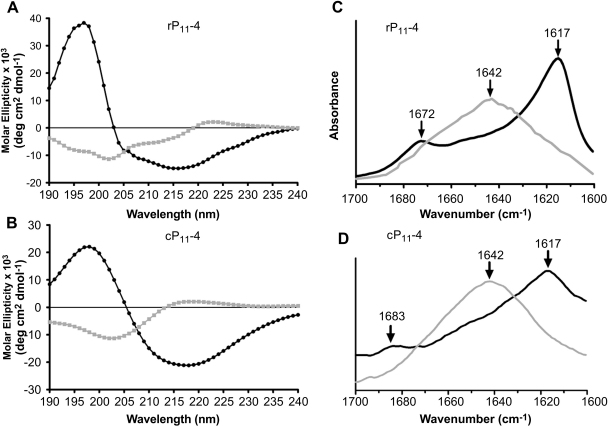
Secondary structure analysis of rP_11_-4 and cP_11_-4. Circular dichroism analysis of (A) rP_11_-4 and (B) cP_11_-4 displayed as molar ellipticity showed similar transitions from β-sheet to random coil upon pH switching from 7.4 () to 9 () respectively. FTIR spectra of (C) rP_11_-4 and (D) cP_11_-4 showing maxima at 1617 cm^−1^ indicating β-sheet structure and weaker maxima at 1672 and 1683 cm^−1^ respectively, confirming an antiparallel conformation. Broad maxima at 1642 cm^−1^ are indicative of random coil for both rP_11_-4 and cP_11_-4.

We examined the secondary structure of rP_11_-4 using CD and FTIR as a function of pH. Solutions of peptide were prepared at a concentration of 10 mg/ml in 140 mm NaCl at pH 7.4 and pH 9, and the samples were then diluted to 100 μm in water with the pH being reconfirmed. The CD spectra for rP_11_-4 at pH 7.4 and pH 9 are shown in Fig. 7A and these spectral data correlate well with that obtained for cP_11_-4 in Fig. 7B. An increase in pH lead to irregular structure and random coil conformation with characteristic negative minimum at ca. 200 nm and positive maximum at 218 nm. On lowering the pH to 7.4, a conformational change occurred characteristic of antiparallel β-sheet formation with negative minimum at 218 nm and strong positive maximum at 198 nm.

FTIR analysis of rP_11_-4 further supported CD data showing that at pH 7.4 a strong peak was observed at 1617 cm^−1^ and a weak peak at 1672 cm^−1^ which confirmed the antiparallel β-sheet configuration of rP_11_-4. Upon increasing the pH to 9, a random coil configuration was adopted and confirmed by the broad peak at 1642 cm^−1^. The FTIR spectra of rP_11_-4 are shown in Fig. 7C and those of cP_11_-4 which showed similar transitions in Fig. 7D.

### 3.12 Cytocompatibility of rP_11_-4

rP_11_-4 samples were plated onto nutrient and heated blood agar, and Sabouraud’s dextrose medium to check for potential microbial growth. No growth of bacteria or fungi was observed on any of the plates indicating that the filtration protocol eliminated microbial contaminants.

**Fig. 8:**
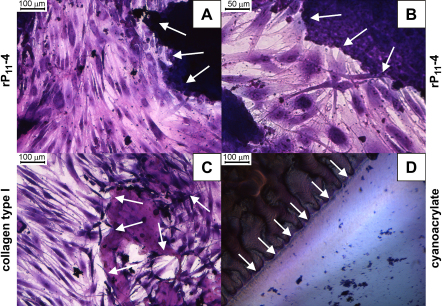
Contact cytotoxicity assay of rP_11_-4 at pH 7.4, 30 mg/ml in DMEM plus supplements following 48 h incubation with primary human dermal fibroblasts. (A–B) rP_11_-4, (C)collagen type I (negative control) and (D) cyanoacrylate (positive control). All images at ×200 magnification, except B which is ×400 magnification. White arrows indicate the periphery of the material with which the cells grew in contact.

rP_11_-4 was prepared in DMEM plus supplements at a concentration of 30 mg/ml at pH 7.4. Contact cytotoxicity assays were used to assess the cytocompatibility of rP_11_-4 with primary human dermal fibroblasts. This enabled preliminary assessment of any toxicity profile associated with rP_11_-4. It was found, by microscopic examination, that primary human dermal fibroblasts grew up to and in contact with rP_11_-4 with no evidence of cytotoxicity (Fig. 8A–B). There were no apparent changes in cellular morphology nor were zones of cell lysis visible. This was comparable to type I collagen (negative control), which also showed no evidence of cytotoxicity (Fig. 8C). By contrast cyanoacrylate (positive control) caused cell lysis and agglomerated cell remnants were seen (Fig. 8D).

## 4 Discussion

There are two broad strategies for the production of self-assembling peptides: chemical synthesis and recombinant production by transgenic organisms ranging from bacteria and fungi to plants and animals. The use of microbial ‘biofactories’ for protein synthesis is widely employed in industry owing to their ease of use, robustness and low costs. Such recombinant systems have the potential for large scale production of peptides and proteins, although there are significant challenges for the production and efficient purification of short (10–30 amino acids) self-assembling peptides.

The Gram-negative bacterium *E. coli* is an attractive system for heterologous protein production due to its ability to grow rapidly to high cell density on inexpensive substrates and excellent genetic characterisation. The large scale production of insulin by Eli Lilly (USA) and bovine growth hormone by Monsanto Corporation attest to the versatility and economic potential of *E. coli*-based therapeutic protein production [25]. Recombinant protein expression using *E. coli* as host is frequently associated with the formation of intracellular protein aggregates as inclusion bodies. For most protein production systems this is undesirable as recovery of functional protein from inclusion bodies requires complex refolding with variable success. For peptides however, this is not an issue and inclusion body recovery formed the basis of a method involving tandem peptide repeats fused to KSI with a 6HIS tag for purification and intervening Met residues for CNBr cleavage [26]. This approach was used in a previous study [18] that demonstrated the peptide coding sequence for P_11_-4 could be successfully expressed as trimer tandem repeats in the pET31b expression system as a KSI-P_11_-4(3)-6HIS construct, with fusion protein yields of 2.5 g/L. Following CNBr cleavage, the recombinant P_11_-4 (rP_11_-4) behaved similarly to the chemically synthesised counterpart, cP_11_-4. The peptide yield obtained was the largest reported at that time of 90 mg/L culture. However, the cleaved peptide product was a combination of rP_11_-4, rP_11_-4-hsl and 6His tag peptide requiring rpHPLC to resolve the former two species from the latter. The final rP_11_-4 sample remained a mixture of peptide alone and that with an additional homoserine lactone derived from the intervening Met residue cleavage, resulting in a net charge change from −2 to −1 at pH 7.4. This resulted in self-assembly occurring only at low pH, and not at physiological pH necessary for cell culture and tissue engineering applications.

The present study focussed on enhancing the production of rP_11_ peptides as unimer species, to overcome the unwanted generation of homoserine lactone species, and on the development of a rapid and simple non-chromatographic purification strategy and finally on the characterisation and cytocompatibility of one of the peptide biomaterials, rP_11_-4.

Starting with a pET31b-KSI-P_11_-4(1)-HIS construct the existing C-terminal 6HIS tag was removed from the expressed protein by inserting two termination codons immediately after the P_11_-4 coding sequence by site-directed mutagenesis. Subsequent site-directed mutagenesis of the resulting pET31b-KSI-P_11_-4(1) plasmid allowed the generation of a series of P_11_-family peptide constructs.

In eliminating the affinity tag for protein purification, it was essential that the method adopted for purifying the fusion protein and peptide were simple and resulted in a pure peptide product. Commonly rpHPLC, affinity or exclusion chromatography methods are used to obtain highly pure peptides and proteins, however, resulting peptide yields are reduced and for scale-up applications the costs associated with these purification methods are high. These considerations led us to develop a more cost-effective high yield purification strategy. *E. coli* cells were lysed by a detergent lysis method and the insoluble, inclusion body fraction recovered by centrifugation. This pellet which comprised predominantly the KSI-rP_11_-4 fusion protein was treated with CNBr and cleavage was at >90% efficient. The sample was again centrifuged to recover the supernatant which was adjusted to pH 9 then filtered (0.22 μm) and subjected to 5 cycles of lyophilisation and water wash steps. While only five wash steps were performed it seems likely that further wash steps may increase the purity of the resulting peptide. SDS-PAGE analysis confirmed that the resulting rP_11_-4 showed no contamination with fusion protein.

Likewise the same simple cleavage, wash, filtration and lyophilisation strategy was shown to be suitable for purification of P_11_-2, P_11_-9, P_11_-13, P_11_-14, P_11_-20 and P_11_-4+RGD demonstrating the generic nature of this approach for these short self-assembling peptides that display divergent amino acid sequences.

From a theoretical rP_11_-4 peptide yield of 371.2 mg/L, a 55% recovery was achieved of 203 mg/L culture. This exceeds our previous demonstration of 90 mg/L and we believe this now to be the highest reported self-assembling peptide yield to be recovered from a recombinant system, notable as our purification strategy does not involve a chromatographic step. This certainly represents one of the simplest recovery methods reported yet with the highest yield of target peptide. Losses may have occurred in the filtration step, where some self-assembly may have occurred due to a concentration effect and further optimisation of this approach may achieve even higher yields.

Middelberg and co-workers also recently reported a chromatography-free purification system in which homogenized cell pellets were heat treated to 55 °C for 15 min. TEV protease was then added to release the P_11_-2 peptide from the thioredoxin fusion partner. The cleavage reaction was then precipitated by ethanol (75% v/v) in the presence of 250 mm NaCl, centrifuged and the supernatant dried before the peptide was resuspended in water. The overall yield was 40% allowing recovery of 13 mg/L culture, although the culture density was significantly lower than we achieve by auto-induction. An rpHPLC polishing step was still used for buffer exchange of the peptide sample prior to self-assembly analysis [20] whereas in our study we used the peptide directly from the wash and lyophilisation process.

The capacity of rP_11_-4 to form a self-supporting hydrogel was assessed. rP_11_-4 was prepared at the same concentration (10 mg ml^−1^) in cell culture medium (DMEM) at pH 7.4 and it started to form a viscous liquid within minutes. After equilibration for 24 h at 25 °C, it had formed a self-supporting hydrogel. To assess whether rP_11_-4 behaved in a similar pH-responsive manner to cP_11_-4, the pH of rP_11_-4 was increased to pH 9 and a liquid state formed instantaneously confirming that rP_11_-4 behaved in a similar pH-responsive manner to cP_11_-4 becoming unimeric at pH 9.

TEM showed that rP_11_-4 formed fibrils with lengths and widths similar to those reported for cP_11_-4 reported by [13,18,24] and to our previously purified rP_11_-4/rP_11_-4 (hsl) [18]. Circular dichroism analysis suggested that the predominant secondary structure of rP_11_-4 at physiological pH (pH 7.4) was antiparallel β-sheet, as has been observed for rP_11_-4/rP_11_-4 (hsl) [18] and for cP_11_-4 [13]. Upon adjusting to pH 9, a conformational transition to random coil occurred. FTIR analysis also indicated an antiparallel β-sheet conformation at pH 7.4 and a random coil conformation at pH 9. These data indicated that rP_11_-4 behaves similarly to cP_11_-4 [13] and [24].

Cytocompatibility studies were performed, in which primary human dermal fibroblasts were grown up to and in contact with rP_11_-4, collagen I and cyanoacrylate for 48 h. This indicated that rP_11_-4 purified by filtration, lyophilisation and wash steps alone was compatible with growth of these primary human cells.

## 5 Conclusions

We have demonstrated that various P_11_-family β-structure self-assembling peptides can be produced in an *E. coli* expression system. Efficient purification was achieved by CNBr cleavage and a series of simple wash, filtration and lyophilisation steps. For rP_11_-4 which was analysed in greatest detail, this ultimately resulted in a peptide yield of 203 mg/L which we believe to be the highest reported self-assembling peptide yield to date. Microscopic and spectroscopic analysis showed that rP_11_-4 had essentially identical properties to the chemically synthesised counterpart (cP_11_-4). rP_11_-4 self-assembled into a hydrogel in physiological conditions and cytocompatibility studies indicated that rP_11_-4 hydrogel was non-cytotoxic towards primary human dermal fibroblasts. This study demonstrates that rP_11_-4 and other P_11_-series peptides can be produced in a sustainable manner using an *E. coli* expression system resulting in a pure peptide without the need for costly chromatographic purification strategies. This method has great potential for large scale production of self-assembling peptides as scaffolds for tissue engineering and human cell culture studies are underway.

## Figures and Tables

**Table 1:** Composition of auto-induction growth media. Adapted from [19].

Growth medium	ZY			NPSC				MgSO_4_ mm	Trace metals (1000× stock)	Lac^50^		
	Tryptone g/L	Yeast extract g/L	NaCl g/L	NH_4_Cl mm	Na_2_SO_4_ mm	KH_2_PO_4_ mm	Na_2_HPO_4_ mm			Glycerol	Glucose	α-lactose
										% (v/v)	% (w/v)	% (w/v)
LB	10	5	10	50	5	25	25	2	0.2×	0.5	0.05	0.2
8ZY4Lac	80	40	0	50	5	25	25	2	0.2×	2.0	0.20	0.8

**Table 2:** Reported levels of production and yields of self-assembling peptides produced using recombinant systems.

Peptide	Host	Fusion protein yield (mg/L)	Theoretical peptide yield (mg/L)	Peptide purified (mg/L)	Efficiency of peptide purification (%)	Reference
Aβ_11–26_	*Escherichia coli*	80	27	10	37	[27]
RADA	*Ralstonia eutropha*	433	116	10.1	8.7	[28]
SA2	*Escherichia coli*	300	30	30	100	[29]
P_11_−2	*Escherichia coli*	85	6.8	2.63	38.7	[17]
P_11_−2	*Escherichia coli*	330	32.1	13	40.5	[20]
P_11_−4	*Escherichia coli*	2500	530	90	17	[18]
P_11_−2	*Pichia pastoris*	800–1000	45	–	–	[30]
P_11_−14	*Pichia pastoris*	600–700	75	–	–	[30]
P_11_−4	*Escherichia coli*	4640	371.2	203	59.5	This study

## Appendix

Figures with essential color discrimination. Figs. 6 and 8 in this article are difficult to interpret in black and white. The full color images can be found in the online version, at doi:10.1016/j.biomaterials.2010.08.051.
